# Hospital and Institutional News

**Published:** 1920-06-05

**Authors:** 


### HOSPITAL AND INSTITUTIONAL NEWS.

### A SURGEON'S WORK FOR HOSPITALS.

The retirement of Sir John Bland-Sutton from the post of senior surgeon to the Middlesex Hospital, after thirty-four years' work, is a regrettable reminder of the passage of time. For he has been not only a distinguished surgeon, but an institutional worker, who has added not only to the surgical reputation, but to the departments and buildings of the hospital. The Bland-Sutton Institute of Pathology will be a permanent memorial to this side of his work. The hospital and the medical school are placed thereby permanently in his debt. Sir John Bland-Sutton has been, in fact, a hospital man in the best sense. The patient, the medical student, the research worker, have severally gained from his energies. He has been an active supporter of hospitals for the middle classes, and both as a member of the medical board of reference and as a visiting surgeon at Fitzroy House has encouraged the Home Hospital movement, and helped to keep a high standard for the uncommercial "nursing home." We have no doubt that such extra leisure as may come to him will be generously used, as before, for the benefit of hospital work.

### MEDICAL HONORARIA.

It is satisfactory to note that a member of the cheaper and more widely-read press has, in contrast with its old-time custom of casting frequent but ill-considered criticism on the workings of voluntary hospital staffs, given space to an appeal for their more reasonable remuneration. It is a subject to which we have repeatedly drawn attention, and in some quarters reforms have been made. It is an undeniable fact that in this country the younger surgeons and physicians are compelled to exist on starvation wages at a time when both their hospitals and the country expect them to produce a maximum output of highly skilled work on behalf of the public. Among these men are the pick of the future profession. Governors of hospitals well know the present situation, but the public, more or less grateful, as it should be, does not. Such men have neither time nor encouragement towards private practice, and yet they are expected to give their best for a remuneration which is less than that of the average labourer. We realise that the hospital staff is the only justifiable path to Harley Street, and to a certain extent it is best that the competitive and other difficulties thereon encountered should be severe. But nevertheless, the present situation tends to give a grossly unfair advantage to the fortunate possessor of an independent income. Work produced under almost "sweated" conditions can in exceptional cases only provide that advance and encouragement in medical science which are a present-day national necessity. Against the situation at it now is, we make an emphatic protest.

### RED CROSS AMBULANCES AND HEALTH SERVICES.

Sir Arthur Stanley points out that the report issued by the Consultative Council of the Ministry of Health embodies recommendations which will necessarily entail a considerable increase in the work of transport of the sick and injured. He informs us that the Joint Council of the British Red Cross Society and the Order of St. John have been able to anticipate this demand by establishing throughout the country over three hundred motor-ambulance stations, and their usefulness is proved by the fact that more than 12,000 cases have been transported. The administration of the ambulances has been delegated through county directors to local organisations or Public Health authorities as circumstances directed, but central control of the service is maintained at the headquarters of the Red Cross. The Joint Council are thus in a position to develop and extend the scheme on such lines as may prove most desirable to meet the needs of a co-ordinated health service.

### MEDICAL BENEVOLENT FUND.

Her Majesty the Queen has given her patronage to the Royal Medical Benevolent Fund Guild, which itself dates from 1909, although the parent fund as originally instituted was founded in 1836. The Guild is managed entirely by ladies, of whom Lady Fripp is the Chairman of the General Purposes Committee and Dame Mary Scharlieb the Hon. Treasurer. The object of the Guild is to assist distressed members of the medical profession and their wives, widows and families. In giving personal sympathy and help the members of the Guild hope to get into touch with all the trouble of doctors who cannot make their wants known in the general way. We give this preliminary notice, although the matinée will not take place until the autumn and the venue will then be His Majesty's Theatre. Communications with reference to the Fund may be sent to Lady Fripp, 19 Portland Place, W. 1.

### INDUSTRIAL DISEASE.

There are at the moment so many vital matters under consideration in the hospital world and so many urgent appeals for help towards mere upkeep that, as we note in our leaders to-day and have before ventured, the endowment of professional chairs deserves somewhat secondary consideration. Nevertheless the latest research suggestion is worthy of general appreciation. Industrial medicine is a branch to which Government assistance should undoubtedly be given and the initial idea of endowing with £20,000 a chair in this subject should receive full official sympathy. Generally speaking knowledge of industrial medicine is small, and this ignorance costs the State millions per annum in sick benefits, etc. Isolated investigations have certainly been made, but their success only goes to prove how much more might be done if any systematic and co-ordinated effort were made. It is not a matter to be left to private subscriptions alone. Both employers and the State in following their plain duty stand to gain greatly in this matter, and it should be urged that a carefully thought out scheme be devised and a central body created specially to tackle the many problems, be they slight or serious, of industrial disease and welfare.

### ANTHRAX IN SHAVING BRUSHES.

In spite of the amusing letters which followed the recent suggestion in *The Times* that every new shaving-brush should be boiled for 30 minutes and then soaked in antiseptic before use, the writer of this warning has in no sense exaggerated the danger which lies in an anthrax infection, and, although his knowledge of the precise anatomy of the average shaving-brush and the dire results of boiling upon its structure may have been a little defective, yet the moral he would teach is essentially sound. If his critics had ever encountered a case of true anthrax infection, they would be less ready to scoff. At least let them use a brush which *can* be boiled and kept in antiseptic, and these articles have little claim to "great costliness" if they cannot with impunity be so treated—and in any case let them remember that fibre brushes are cleaner if only from the fact that "it is the hair and the hair alone which is dangerous."

### A GOOD DEAL FOR BART'S.

The Governors of St. Bartholomew's Hospital, with the consent of the Charity Commissioners, have decided to sell some of their property in the City of London. The sale is by private treaty, and the price to be paid is £26,000, the purchasers paying all the costs of the transference of the property. This is described in the schedule as a piece of land situate in the parish of St. Sepulchre Without, in the County of London, together with the warehouses thereon and known as Nos. 91 and 93 Charterhouse Street, subject to and with the benefit of an indenture of lease for the term of fourteen years from December 25, 1913. Perhaps this is some of the property left for the benefit of the old hospital and priory by Rahere, the founder. It is unfortunate that the governors have to sell any of their property. There is the consolation, however, of knowing that the governors have made a good bargain, and have taken advantage of the existing state of the property market.

### MR. THOMAS RYAN.

The resignation of Mr. Thomas Ryan from the secretaryship of St. Mary's Hospital, which will take effect later in the year, will remove from the roll of hospital secretaries a personality which has exercised an influence on the general work of the voluntary system for a period of forty years. Mr. Ryan's first experience of hospital life was in the service of the Seamen's Hospital Society when Sir Henry Burdett was secretary of that institution. There Mr. Ryan laid the foundation of his future position in the hospital world and acquired that knowledge of hospital administration and finance which served him so well in later years. When the secretaryship of Queen Charlotte's Hospital became vacant in 1880 Mr. Ryan was appointed to that office. There he did really excellent work. He had the advantage of having as treasurer Mr. Alfred C. de Rothschild, and the financial position of that institution improved very materially. In the first Jubilee year, 1887, on the resignation of Mr. P. J. Michelli from the secretaryship of St. Mary's, Paddington, on his appointment as secretary of the Seamen's Hospital Society, Mr. Ryan was appointed to succeed him at the former Institution. At that time the question of the acquisition of the premises abutting on to Praed Street was engaging the attention of St. Mary's Hospital Committee, and very soon after Mr. Ryan's appointment the site was acquired upon which now stands the "Clarence Memorial Wing." It may be that the project was premature, for the buildings remained unoccupied for many years owing to the lack of funds to maintain them. However, later on the development of pathological research enabled a portion of the buildings to be utilised, and a very fortunate legacy which had been left in reversion to St. Mary's many years before came to fruition and brighter prospects opened for the Institution. During Mr. Ryan's tenure of office the old open Board which existed when he was appointed has been done away with and a Board of Management appointed in its stead. Among Mr. Ryan's earlier activities was the management of the Bischoffscheim Ambulances for street accidents in the Metropolis. They were the forerunners of the present L.C.C. ambulances, of which to-day they form a useful adjunct. Mr. Ryan has taken part in hospital matters outside his own institution. He was President of the Hospital Officers' Club on several occasions, and the Club prospered under his guidance. He also held office as President of the Incorporated Society of Hospital Officers. The vacant secretaryship of St. Mary's Hospital has been advertised, and the salary offered is £800 a year, rising by increments of £25 to £1,000. Candidates must be between the ages of 30 and 42. Previous hospital experience, for some reason, is not required.

### HEALTH SCHEME FOR WALES.

The Welsh Consultative Council on Medical and Allied Services, appointed by the Ministry of Health, has now issued its report. The reference was to consider and make recommendations as to a scheme for systematical provision in Wales of such forms of medical and allied services as should be available for the inhabitants of a given area. Naturally such a scheme would have to be considered in relation to a comprehensive policy for the extension and development of health services, and why the Consultative Council should have sat before such policy had been determined it is not easy to understand. Subject to this, however, much of the work of the Welsh Council cannot fail to be of future value. The question of health visiting naturally takes a leading place, and the value of the male health visitor, for nursing men in early nerve cases, for attendance at male venereal clinics or ablution centres, and for other service, is not forgotten. The Council consider that the minimum number of qualified female health visitors (for carrying out in a more satisfactory manner the limited duties at present performed by such officers in connection with infant-welfare work and school medical inspection)

should be one per 1,000 houses in the larger centres of population and one per 500 to 750 houses in the smaller towns and rural districts.

### ONE DOCTOR FOR FOUR HUNDRED HOMES.

Acting with the assistance of the various officers whose duties are outlined in the report, one doctor should, as a general rule, be available for 400 homes in urban communities, or for 300 homes in rural. The unequal distribution of the population adds to the complexity of the problem, especially as to the position of specialists and consultants, and it is suggested that this side of the question should be approached on national rather than on local lines. The Council are of opinion that all health institutions, including voluntary hospitals, should form part of the future public services. Until the general question of health administration in Wales has been more fully considered it is thought desirable to postpone the formulation of proposals as to the highest grade of institutions that will form the coping-stone of institutional organisation for Wales. Subject to this the Welsh Council suggest a scheme very similar to that of the English Consultative Council, which was published at about the same time. For instance, the "Central Institute for General Medical and Surgical Purposes" would correspond to the Health Centre of the English report. The Welsh Council, however, enter into greater detail as to various clinics, convalescent treatment, asylums, infectious disease hospitals, tuberculosis sanatoria, institutions for the blind and the like. The report, which includes the results of the visits of sub-committees to typical areas in Wales, is excellently arranged, and should be of material help to the Minister of Health.

### FINANCIAL TROUBLES OF CHILDREN'S HOSPITALS.

Two South London hospitals for children are feeling severely the increased cost of commodities. The Evelina Hospital for Sick Children in Southwark Bridge Road, situated in one of the poorest and most densely populated districts, finds itself with an annual expenditure of about £12,000; only £4,000 from investments, and the rest to be raised by subscriptions. There is a deficit of £4,000, and an attempt is to be made to raise £10,000 to clear this off and to cover the cost of repainting the building, work long overdue, but delayed by the war. The Committee at the Belgrave Hospital for Children, Clapham Road, finds itself in similar straits. The babies' ward has always been a source of anxiety, and was closed the whole of last year owing to lack of nurses, but arrangements have been made to increase the nursing staff, and it will shortly be reopened. The expenditure was £1,118 more than in the previous year, upwards of £500 having been spent in cleaning and redecorating. Funds are very badly needed.

### CONSUMPTION IN THE PRINTING TRADE.

The National Society of Operative Printers and Assistants has acquired Temple Hall, near Wellesborough, in Leicestershire, for a sanatorium, to be known as the Natsopa Memorial Home for consumptive printers, and for convalescent and aged printers and their wives. It is a War Memorial to the 341 members of the Society who fell in the War, and the Hall, which consists of a farm with 310 acres, was opened on Friday last week, as the beginning of the scheme. The idea sprang from the fertile brain of Mr. G. A. Isaacs, general secretary of the Society. It has been supported by the big newspaper proprietors, who have collectively given the chief part of the £8,638 so far raised. Mr. E. J. Williams is the architect, and we shall hope to publish the plans eventually. Lord Burnham, one of the speakers, emphasised the need for extending sanatorium treatment. We would wish to say a word upon the dangers from tuberculosis to which the printer is exposed. In spite of the Factory Acts, he lives and works in a dry atmosphere of ink and dust. In spite of trade regulations which, on both sides, have to be, from the conditions of the work, interpreted generously, he sometimes has to work very late, nor is his gardening, for many printers are enthusiastic gardeners, a sufficient corrective of this impaired air and these periodic extensions of work. No wonder, then, that he is apt to fall a victim to tuberculosis. Those readers who are also hospital men will be intelligently sympathetic, and, we hope, feel disposed to add to the fund now started for the Natsopa Sanatorium and Home.

### WHAT IS LABOUR DOING?

"What is labour doing?" This question was asked at the annual meeting of the Governors of the East Sussex Hospital at Hastings. No direct answer was forthcoming, "but it would not be far wrong," writes a correspondent, "to say that the support of labour, not in Hastings only, is not commensurate with its wages of to-day." The matter provides a sharp argument to the advocates of State control of hospitals and allied institutions, because then all classes would be called upon to contribute through the tax office, and the middle and richer classes, who have brought the hospitals to their present state of efficiency, would pay only an equal share with those of the industrial classes, instead of the larger share which they give now. Another experience not peculiar to Hastings is how quickly the hospitals are forgotten when people are well, and how, when ill, their first thoughts fly to the skill of the doctors and the nurses at the hospitals. The truth of the matter is that too much is being spent on amusements of all sorts, to the detriment of public health services. For the present, propaganda work must be resorted to seriously; some means must be devised to bring the calls of the hospitals home to the "new rich." This criticism is not new, but its repetition, by a layman, is none the less useful.

### HOW TO MAKE A BUILDING RAT-PROOF.

Dr. W. J. Howarth, Medical Officer of Health for the City of London, is, to use a common phrase, "rough on rats." He has attempted to rid the City of the pests, and probably his latest report may further tend to exterminate them. Dr. Howarth points out that there are two types to be found in the City, the black rat and the brown. These differ considerably in their habits. The black variety seldom enters premises by drains, and does not, as a rule, burrow. It generally establishes itself in walls, between floors and ceilings, and roofs. An expert climber, it not only gets to roofs and window-ledges by means of stack wires attached to the walls, but travels long distances on overhead electric and telephone wires. These rats may be seen on a bright night crossing overhead from street to street. The brown rat is always to be found in sewers and drains, generally entering a building by a defective drain or an unprotected area window. It is also a good climber, but not a Blondin like the black rat. Frequently a brown rat burrows from a defective drain or sewer under the pathway, and works its way along until it finds a weak spot in a basement wall or the brick-work of a coal-shoot, where it gnaws an entrance. It often breeds under the pavement, and does great damage. Dr. Howarth proceeds to show how they can be reduced in numbers. To rid buildings of rats the problem resolves itself into the removal of internal structural defects, so as to ensure that walls, rooms, floors, and ceilings shall furnish no nesting-places, and the storing of all food in rat-proof receptacles. An efficiently rat-proofed building should provide no means for breeding or shelter, and the walls should be resistent to the burrowing or gnawing activities of the rats. In the City, however, it is only the more modern buildings which are so satisfactorily constructed. The old buildings appear to furnish rats with every convenience.

### A COUNTRY BRANCH OR THE LEICESTER ROYAL INFIRMARY.

THE Committee of the Leicester and Leicestershire Disabled Warriors' Fund has approved of a proposal to hand over to the Leicester Royal Infirmary the Leicester Frith Home, established in connection with the Mayor of Leicester's fund for the treatment of neurasthenic and shell-shocked soldiers. It is now intimated that the type of cases admitted to the Home has entirely changed, and numbers have dwindled so much that the Committee has thought it necessary and right to make representation to the Ministry of Pensions that it would not be proper to allow it to use the Home beyond March 1 next, and that larger institutions would be more satisfactory for the treatment of the type of case now received in the Home, a view with which the Director-General of the Ministry agrees. The intention of the Governors of Leicester Infirmary is to use the Home as an auxiliary hospital, thus making the available accommodation of the Infirmary more elastic. The Home will accommodate about sixty patients, and while adding considerably to the cost of the Infirmary, cannot fail to be of the greatest service in meeting the increasing demands.

### AN EXTENSION OF THE UNIFORM SYSTEM.

THE movement in favour of sectional subscriptions is meeting with success at Bradford, where, chiefly from employers, a sum of £18,000 a year is hoped for from this newly developed source. The gain already made, and the scheme is only in process, shows already that subscribers, who last year gave £2,780 to the Bradford Royal Infirmary, have given or promised £6,670, an improvement of some 300 per cent. The aim, in fact, is to raise £3 where £1 has been raised hitherto, and of course to increase the number of subscribers. An excellent article lately appeared in the *Bradford Weekly Telegraph*, which not only marshalled clearly the figures relating to the Royal Infirmary there, but also gave instructive comparisons between the income from investments, and to some extent from workmen's subscriptions at Bradford, Leeds, Manchester, Sheffield, and Burnley. We welcome this instalment of co-operative statistics. We have always held that these should not be merely consulted in "Burdett," but made common knowledge by intelligent editors. Besides, the need of the present is not merely for a Uniform System of Accounts, but for a uniform system of hospital statistics generally. To get this the cost per occupied bed, for instance, must be determined by a common method of computation. If Sir Napier Burnett's survey leads to this beneficent result, it will be a great gain. It is the end to work for.

### THE BOURNEMOUTH "BOBBIES."

THE police of Bournemouth are very sympathetic to their hospital, and recently they organised a football match, which was played by teams representing Bournemouth Police *versus* Boscombe Police. An old friend of the hospital gave a silver cup and medals for competition, and the result was a good sporting game, and, best of all, a handsome cheque for £281 9s. 8d. for the institution. Bournemouth has not the large football following of enthusiasts such as large industrial towns and cities have, but the above effort is an excellent example of practical help from the police. It is hoped to make the football match an annual affair, and the police have another scheme in hand to help the finances of the Royal Victoria and West Hants Hospital, Bournemouth. We congratulate the police and their hospital.

### THIS WEEK'S DRUG MARKET.

THERE is no improvement in the drug market, and while business is not actually at a standstill it is extremely quiet, especially so far as the home trade is concerned. A fair amount of orders continues to come in from some of the Colonial markets, but this is not sufficient to prevent prices from sagging. The opinion is now more prevalent than ever that the easier tendency of prices will continue until a lower level is reached, at which prices will be more stable. Sharp declines in values generally are hardly expected, although here and there articles may drop in value more substantially. On the other hand, there will no doubt be a few special cases in which prices will incline upwards owing to specific cases. Makers of calomel, corrosive sublimate and other salts of mercury have reduced their prices in consequence of the sharp decline in the value of quicksilver. Cod-liver oil is still extremely quiet; in fact, the same may be said of the market for most commodities.

